# Have You Seen My Teeth? A Case with an Extraordinary Radiologic Finding

**DOI:** 10.1055/s-0036-1580707

**Published:** 2016-03-14

**Authors:** Serdar Evman, Yelda Tezel, Melis Demirag Evman, Çagatay Tezel

**Affiliations:** 1 Department of Thoracic Surgery, Sureyyapasa Chest Diseases and Thoracic Surgery Training and Research Hospital, Istanbul, Turkey; 2 Department of Pulmonology, Haydarpasa Numune Training and Research Hospital, Istanbul, Turkey; 3 Department of Otorhinolaryngology, Marmara University Hospital, Istanbul, Turkey

**Keywords:** bronchoscopy, computed tomography, foreign body, trachea

## Abstract

A 55-year-old man was admitted to the emergency department with severe dyspnea and hoarseness, starting immediately after a hypotensive syncope attack at home. Pulmonary auscultation revealed generalized stridor and right-sided wheezing, with no finding in the upper airway on direct laryngoscopy. Chest X-ray and computed thorax tomography revealed a high-density foreign body on the carina, causing partial obstruction in the right main bronchus. The fractured dental plate, probably aspirated during the syncope attack, was successfully removed by rigid bronchoscopy. The postoperative period was uneventful and the patient was discharged on the same day. Rapid physical and radiologic examination of patients with severe acute dyspnea is vital for determining the treatment modality and preventing any potential mortality.

Endobronchial foreign body aspiration is rare in the adult population with normal cognitive and mental functions. 1 2 According to the nature of the aspirated object and the baseline health status of the patient, it can even be fatal, especially for pediatric patients. 2 Rigid bronchoscopy still plays the main role for the definite diagnosis and removal of the aspirated material.

## Case Report

A 55-year-old man was admitted to the emergency department with acute onset of dyspnea following a hypotensive syncope attack at home. Physical examination revealed no hypotension or fever, Glasgow Coma Score of 15, and severe dyspnea with diffuse stridor and hypoxemia (arterial PaO _2_ = 59 mm Hg, SaO _2_ = 83%) on admission. There was no history of any known respiratory diseases.

**Fig. 1:**
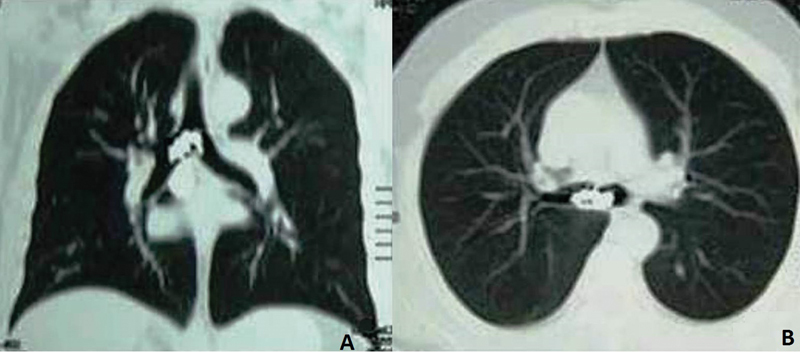
(A, B) Chest tomography showing aspirated foreign body at the carina, extending into right main bronchus.

**Fig. 2:**
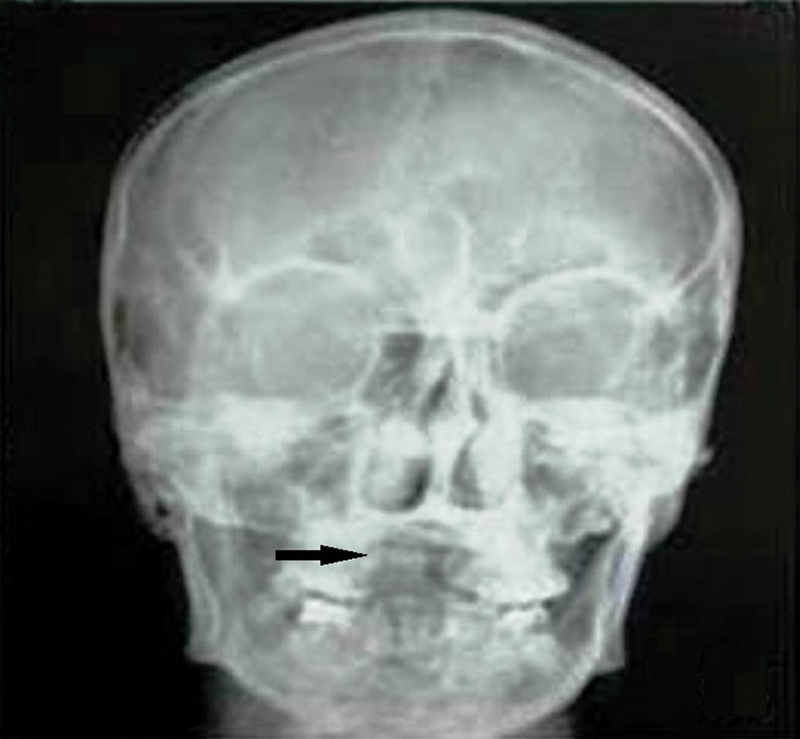
Skull X-ray demonstrating absence of upper incisor teeth (arrow).

**Fig. 3:**
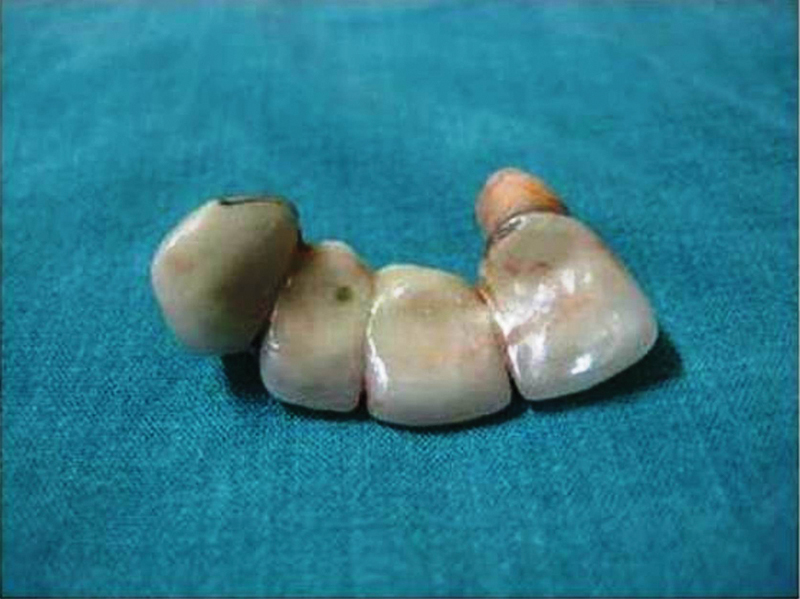
Removed dental plate.

Cranial and thoracic computed tomography revealed no pathologic sign of traumatic cranium fracture or intracranial hemorrhage but confirmed an opaque foreign body localized at the carina partially obstructing the right main bronchus ( Figs. 1A, B ). Posteroanterior skull radiograph confirmed the missing part of a dental prosthesis ( Fig. 2 ) that probably fragmented during the syncope attack, which was immediately removed under rigid bronchoscopy ( Fig. 3 ). The etiology of the hypotensive episode could not be established. The patient was discharged on the same day and was referred to the cardiology outpatient clinic for further investigation.

## Discussion

A wide variety of aspirated items, which may demonstrate regional, seasonal, or cultural dispersion, are published in the literature. 1 2 3 Craniofacial trauma, medical interventions like endotracheal intubation, or conditions linked to altered consciousness such as mental retardation, dementia, or intoxication are among the known risk factors. 2 4 5 The aspiration of prostheses or teeth during trauma, accidents, and dental procedures has been reported in the literature. 4 5 6 Tooth aspiration during medical interventions such as endotracheal intubation has also been described. 7

The right main bronchus, like in our case, is the most common site for aspiration, owing to its anatomical position and the slight angulation of the trachea toward the right side. The most helpful diagnostic methods are the objective complaint of witnessed aspiration and the physical examination of the patient, but radiologic assessment is essential and should be performed if there is any suspicion of foreign body aspiration. Rigid bronchoscopy is the long-established gold standard treatment of choice. 3 5

The acute onset of dyspnea following trauma may be caused by an intracranial hemorrhage, pneumothorax, or a foreign body occluding the airway. Rapid physical examination of patients with severe acute dyspnea is vital for preventing morbidity and mortality.
